# Saliva microRNA Biomarkers of Cumulative Concussion

**DOI:** 10.3390/ijms21207758

**Published:** 2020-10-20

**Authors:** Steven D. Hicks, Robert P. Olympia, Cayce Onks, Raymond Y. Kim, Kevin J. Zhen, Gregory Fedorchak, Samantha DeVita, Aakanksha Rangnekar, Matthew Heller, Hallie Zwibel, Chuck Monteith, Zofia Gagnon, Callan D. McLoughlin, Jason Randall, Miguel Madeira, Thomas R. Campbell, Elise Fengler, Michael N. Dretsch, Christopher Neville, Frank A. Middleton

**Affiliations:** 1 Department of Pediatrics, Penn State College of Medicine, Hershey, PA 17033, USA; kzhen@pennstatehealth.psu.edu; 2 Department of Emergency Medicine, Penn State College of Medicine, Hershey, PA 17033, USA; rolympia@pennstatehealth.psu.edu; 3 Department of Family Medicine, Penn State College of Medicine, Hershey, PA 17033, USA; conks@pennstatehealth.psu.edu; 4 Department of Orthopedics and Rehabilitation, Penn State College of Medicine, Hershey, PA 17033, USA; rkim@pennstatehealth.psu.edu; 5 Quadrant Biosciences, Institute for Human Performance, Syracuse, NY 13210, USA; gregory.fedorchak@quadrantbiosciences.com (G.F.); samantha.devita@quadrantbiosciences.com (S.D.); aakanksha.rangnekar@quadrantbiosciences.com (A.R.); 6 Department of Family Medicine, New York Institute of Technology College of Osteopathic Medicine, Old Westbury, NY 11568, USA; mheller@nyit.edu (M.H.); hzwibel@nyit.edu (H.Z.); 7 Department of Athletic Training, Colgate University, Hamilton, NY 13346, USA; cmonteith@colgate.edu; 8 School of Science, Marist College, Poughkeepsie, NY 12601, USA; zofia.gagnon@marist.edu (Z.G.); cmcloughlin8864@gmail.com (C.D.M.); jrandall2323@gmail.com (J.R.); miguelmaymone@gmail.com (M.M.); 9 School of Rehabilitation Sciences, Old Dominion University, Norfolk, VA 23529, USA; tcamp012@odu.edu; 10 Department of Exercise Science, Syracuse University, Syracuse, NY 13210, USA; fenglere@chc.edu; 11 US Army Medical Research Directorate-West, Walter Reed Army Institute of Research, Joint Base Lewis-McChord, WA 98433, USA; michael.n.dretsch.mil@mail.mil; 12 Department of PT Education, Orthopedics, and Neuroscience, SUNY Upstate Medical University, Syracuse, NY 13210, USA; nevillec@upstate.edu; 13 Department of Neuroscience and Physiology, SUNY Upstate Medical University, Syracuse, NY 13210, USA; middletf@upstate.edu

**Keywords:** saliva, microRNA, sports-related concussion, mild traumatic brain injury, biomarker

## Abstract

Recurrent concussions increase risk for persistent post-concussion symptoms, and may lead to chronic neurocognitive deficits. Little is known about the molecular pathways that contribute to persistent concussion symptoms. We hypothesized that salivary measurement of microribonucleic acids (miRNAs), a class of epitranscriptional molecules implicated in concussion pathophysiology, would provide insights about the molecular cascade resulting from recurrent concussions. This hypothesis was tested in a case-control study involving 13 former professional football athletes with a history of recurrent concussion, and 18 age/sex-matched peers. Molecules of interest were further validated in a cross-sectional study of 310 younger individuals with a history of no concussion (*n* = 230), a single concussion (*n* = 56), or recurrent concussions (*n* = 24). There was no difference in neurocognitive performance between the former professional athletes and their peers, or among younger individuals with varying concussion exposures. However, younger individuals without prior concussion outperformed peers with prior concussion on three balance assessments. Twenty salivary miRNAs differed (adj. *p* < 0.05) between former professional athletes and their peers. Two of these (miR-28-3p and miR-339-3p) demonstrated relationships (*p* < 0.05) with the number of prior concussions reported by younger individuals. miR-28-3p and miR-339-5p may play a role in the pathophysiologic mechanism involved in cumulative concussion effects.

## 1. Introduction

Concussion is a type of traumatic brain injury that results from sudden acceleration/deceleration of the head, causing brief impairment of consciousness, strength, and memory [1]. Increased awareness of concussion, and proper implementation of diagnosis guidelines have led to steadily increasing rates of sports-related concussions among both collegiate and professional athletes [1,2,3]. Athletes who suffer a concussion typically experience a constellation of symptoms, including headache, dizziness, fatigue, and emotional dysregulation [4]. The character and duration of concussion symptoms can vary widely between athletes. Those who experience recurrent concussions are more likely to suffer loss of consciousness and prolonged symptoms [5].

In rare cases, recurrent concussions can have dire consequences. The compounding effects of multiple concussions may result in second-impact syndrome, a condition characterized by lethal increases in intracranial pressure [6]. Guidelines for the diagnosis and management of sports-related concussion are, to a large extent, aimed at preventing this catastrophic outcome. However, a more commonly observed morbidity of repetitive head trauma is persistent cognitive impairment. Cumulative concussions have been shown to negatively impact an individual’s processing speed [7,8]. In a large proportion of concussed individuals, cognitive impairment (such as memory or visuospatial deficits) can persist for months after injury [9]. In military populations, individuals who report multiple concussions also report prolonged post-concussive symptoms, but often do not have measurable cognitive impairment [10].

Professional American football athletes, who may sustain numerous concussions over the course of a playing career, appear to be at risk for even longer-term cognitive effects. Recurrent concussions have been associated with mild cognitive impairment and clinical depression years later [11,12,13,14]. Collegiate football players also demonstrate slow recovery of neurological function after multiple concussions [15]. Those with a history of concussion display impaired cognitive function on neuropsychological measures, similar to peers with learning disability [16]. Compared with a single concussion, multiple concussions are more likely to impair memory performance among collegiate football players. Notably, however, studies of high school football athletes have not demonstrated a relationship with neurodegenerative disease later in life [17].

Although the clinical consequences that result from recurrent concussions are well described, the pathobiology that mediates these effects remains poorly understood. There is growing evidence that the chronic effects of repetitive traumatic brain injury may result in deposition of hyperphosphorylated tau protein around the small blood vessels of the cerebral cortex, creating neurofibrillary tangles and neurites [18,19]. However, it is unclear how the acute physiology resulting from repetitive concussion would lead to this late, chronic pathologic finding. One explanation is that repetitive head injury may cause persistent activation of microglia, resulting in chronic neuroinflammation [20]. Goldstein and colleagues have demonstrated microvascular injury and perivascular neuroinflammation in a mouse model of concussion with phosphorylated tau pathology [21]. An alternative hypothesis involves dysregulation of the neurometabolic cascade, caused by indiscriminate release of excitatory neurotransmitters and perturbation of the ion pumps that regulate neuronal membrane potential [22]. Regardless of the responsible mechanisms, evidence shows that individuals with a history of concussive injury experience disrupted neural functioning associated with cognition and regulatory processes [23,24].

For these acute pathologic processes to yield chronic impairment in brain function, individual neurons, glia, and astrocytes must undergo persistent dysregulation of cellular processes. Epigenetic mechanisms have the ability to alter cellular function in response to an external insult, and have been implicated in the chronic effects of mild traumatic brain injury [25]. Microribonucleic acids (miRNAs) are small epitranscriptional molecules that are critical to neuronal function [26], and regulate gene expression in response to traumatic brain injury [27,28]. Neurons have the ability to package miRNAs within protective exosomes as an intracellular signaling mechanism [29]. Recent findings suggest that exosomal miRNA levels may provide insights into the pathophysiology through which recurrent concussions lead to chronic symptomology [30].

Previously, we showed that perturbation in miRNA signaling after traumatic brain injury results in overlapping miRNA profiles in cerebrospinal fluid and saliva [31]. Further, we demonstrated that disruptions in salivary miRNA levels can persist for weeks after initial injury [32], and predict the occurrence of persistent post-concussive symptoms [33]. The purpose of the current study was to determine whether non-invasive salivary measurement of miRNAs could provide insights into the pathobiology of repetitive concussions among professional football athletes. We hypothesized that levels of specific salivary miRNAs would be associated with a history of multiple concussive episodes, and that these same miRNAs would demonstrate unique expression profiles among former professional American football athletes. To test these hypotheses, we performed a cross-sectional study of 310 individuals (ages 7–39 years) who provided a detailed concussion history, followed by a case-control study of 31 individuals (including 13 former professional football athletes and 18 age/sex-matched controls), of ages 46–89 years.

## 2. Results

### 2.1. Participant Characteristics

Participants in Group 1 (former professional football athletes and peers) were all male (31/31; 100%), mostly White (14/19; 74%) and had a mean age of 73 (±8) years (Table 1). No individuals reported anxiety, depression, or ADHD. Former professional football athletes differed from controls only in BMI (28 kg/m^2^ vs. 26 kg/m^2^; *p* = 0.004). Both groups reported similar rates of previously diagnosed concussion (*p* > 0.05). However, all of the professional football athletes (*n* = 13) reported undiagnosed concussions, with an average of five concussions sustained during their professional football career (mean career duration 13 ± 2 years). Positions played by the former professional football athletes included quarterback (2), wide receiver (1), offensive/defensive tackle (3), end (2), guard (2), and fullback (1). Former professional football athletes reported an average of 10/22 (range: 3–21) concussion-related symptoms on the PCSS, with a mean symptom severity score of 19 (range: 3–78; 132 possible points). More specifically, difficulty with concentrating (10/13, 77%), and difficulty with balance (9/13, 69%) were the most commonly reported symptoms on the PCSS. There were five former football athletes (38%) who reported short-term memory difficulty, and four who reported cognitive difficulty (31%). None reported self-regulation problems such as impulsivity, apathy, or emotional instability.

Participants in Group 2 (younger individuals with varying concussion history) were mostly male (208/310; 76%), mostly White (224/310; 82%), and had a mean age of 20 (±5) years. Rates of anxiety, depression, and ADHD were similar between individuals with prior concussion and those without prior concussion. Individuals with prior concussion were older, and contained a higher proportion of males. They had sustained an average of 1.5 concussions each (range: 1–7). Individuals with prior concussion reported a similar burden of concussion-related symptoms on the PCSS (mean burden: 2/22 symptoms; mean severity = 5) as those without prior concussion (mean burden: 2/22 symptoms; mean severity = 3).

### 2.2. Functional Measures of Balance, Neurocognition, and Olfaction

In Group 1, former professional football athletes had greater stability than controls while standing in a two-legs eyes-open stance (*p* = 6.2 × 10^−5^) and a two-legs eyes-closed stance (*p* = 0.004; Table 2). There was no difference (*p* > 0.05) between groups in stability during tandem stance eyes open, tandem stance eyes closed, or two-legs eyes open on a foam pad. Former professional football athletes and controls performed similarly on all measures of neurocognition, and did not differ in olfactory performance on the BSIT.

In Group 2, individuals with prior concussion had reduced stability compared to those without prior concussion while standing in tandem stance eyes open (*p* = 0.04), and tandem stance eyes closed (*p* = 0.04). There was no difference between groups in balance errors during the two-legs eyes-open stance, the two-legs eyes-closed stance, or the two-legs eyes-open stance on a foam pad. Individuals with prior concussion performed similarly to those without prior concussion on all neurocognitive measures.

### 2.3. Salivary miRNAs Levels

**Figure 1:**
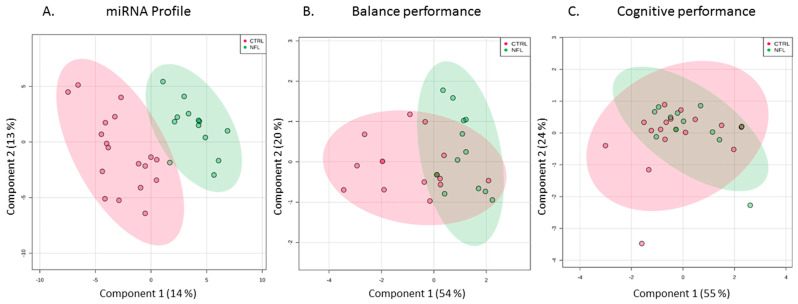
Salivary miRNA profiles differentiate former professional football athletes from peers. Partial least squares discriminant analysis (PLSDA) was applied to individual salivary miRNA profiles (**A**), balance performance measures (**B**), and neurocognitive scores (**C**) among professional athletes in the National Football League (NFL; *n* = 13; green) and control participants (CTRL; *n* = 18; red). The two-dimensional PLSDA plot based on saliva miRNA levels achieved complete separation of groups, while accounting for 27.5% of variance in the miRNA data.

**Figure 2:**
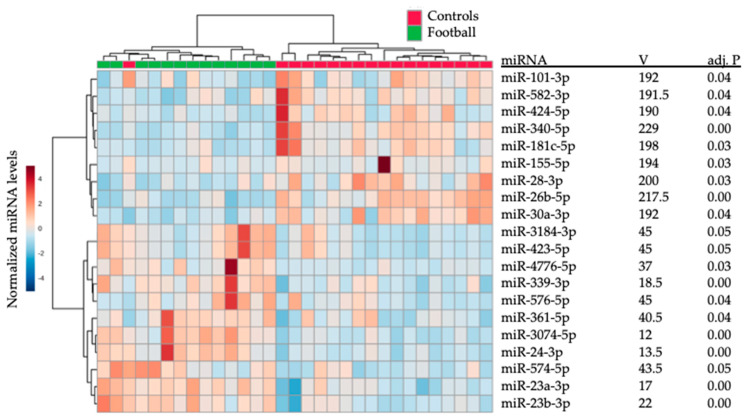
Twenty salivary miRNAs differ between former professional football athletes and peers. The heatmap displays salivary levels of 20 miRNAs with significant differences (adj *p* < 0.05) in salivary expression between former professional football athletes (*n* = 13; green) and control participants (*n* = 18; red). Hierarchical clustering of both participants and miRNAs is based on a Ward clustering algorithm with a Euclidean distance measure. V statistics and adjusted p values on non-parametric analysis of variance are presented for each miRNA.

In Group 1, total salivary miRNA profiles demonstrated superior ability for separating former professional football athletes from controls, when compared with balance or neurocognitive assessments (Figure 1). Salivary miRNA expression achieved complete separation between groups in the two-dimensional model, while accounting for 27.5% of the variance in the data. Among the 264 miRNAs with robust salivary expression, there were 20 with significant differences in expression (adj. *p* < 0.05) between former professional football athletes and controls (Figure 2). Nine miRNAs displayed reduced levels among former professional football athletes, while 11 miRNAs were elevated.

**Figure 3:**
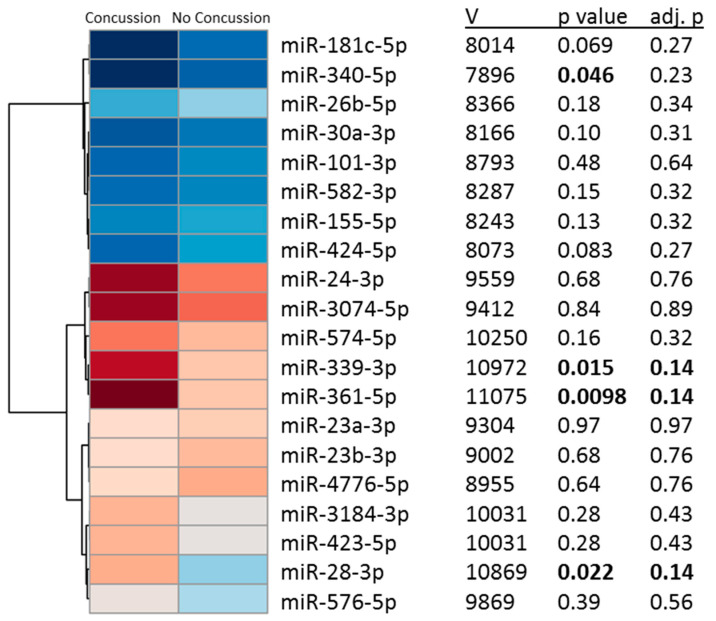
Three miRNA candidates differ among individuals with prior concussion. A non-parametric analysis of variance comparing levels of the 20 miRNA candidates identified in former professional football athletes, identified three miRNAs (miR-339-3p, miR-361-5p, and miR-28-3p) nominal differences (*p* < 0.05; adjusted *p* < 0.15) between individuals with prior concussion, and those without prior concussion. Red denotes increased expression, while blue denotes decreased expression. Notably, for 2/3 miRNAs (miR-339-3p and miR-361-5p), expression levels among individuals with prior concussion mirrored differences observed in former professional football athletes.

In Group 2, non-parametric ANOVA identified nominal differences (*p* < 0.05; adjusted *p* < 0.15) between individuals with prior concussion and those without prior concussion for 3/20 miRNA candidates (miR-339-3p, miR-361-5p, and miR-28-3p; Figure 3). A linear regression analysis assessing the relationship between number of prior concussions and miRNA concentrations (while controlling for age and sex) identified a significant relationship between prior concussions and miR-339-3p (R = 0.21, F = 2.7, *p* =0.02), as well as miR-28-3p (R = 0.40, F = 11.4, *p* < 0.001), but not miR-361-5p (R = 0.19, F = 2.2, *p* = 0.053).

### 2.4. Biologic Functions and Brain Relatedness of miRNA Candidates

DIANA miRPath analysis was used to interrogate putative messenger RNA targets for the three miRNAs (miR-28-3p, miR-339-3p, and miR-361-5p) that had demonstrated significant differences among former professional football athletes (Group 1), and nominal differences among individuals with prior concussion (Group 2). These three miRNAs target 1068 messenger RNAs (Tarbase alignment, adj. *p* < 0.01), demonstrating enrichment for eight Kyoto Encyclopedia of Genes (KEGG) pathways (Table 3). KEGG pathways with potential implications in chronic brain injury and inflammation included extra-cellular matrix interaction (adj. *p* = 2.6 × 10^−7^; 9 genes; 3/3 miRNAs), protein processing in endoplasmic reticulum (adj. *p* = 6.3 × 10^−5^; 25 genes, 3/3 miRNAs), and lysine degradation (adj. *p* = 0.0009; 7 genes; 2/3 miRNAs). Tissues of origin were interrogated for the three candidate miRNAs using the Human miRNA Tissue Atlas. All three miRNAs showed robust expression in both nervous system and gastrointestinal tissues. Levels of miR-361-5p were highest in brain and cerebellum, miR-339-3p was highest in colon and cerebellum, and miR-38-3p was highest in the small intestine and nerves.

## 3. Discussion

In the current study, we identified three miRNAs that demonstrate a potential physiologic relationship with recurrent concussion. Compared with healthy peers, former professional football athletes who reported a history of multiple undiagnosed concussions demonstrated significant differences in levels of these three miRNAs. The three miRNAs also demonstrated differences among younger individuals with prior concussion, when compared to peers without prior concussion. Levels of two of the miRNAs were significantly related to the number of previous concussions, suggesting they may change over time as a result of subsequent concussion(s).

Prior studies have shown that recurrent concussions can lead to chronic impairment in memory, processing speed, and emotional regulation [11,12,13,14]. In the present study, we found no significant differences between former professional football athletes and age/sex-matched peers in neurocognitive performance. Younger individuals with prior concussion also demonstrated no significant difference in neurocognitive abilities from peers without prior concussion. Neurocognitive tests represent semi-objective, clinically available measures that have shown decrements in prior studies of professional athletes with multiple concussions [11]. Sensitivity of neurocognitive testing for detecting concussion-related effects may require baseline testing with annual repeated measures, which were not available in the current study [34]. Alternatively, neurocognitive testing may lack sensitivity due to general neurologic compensatory processes that could mask compromised functioning [35].

Notably, former professional football athletes outperformed peers on several measures of balance, which may be attributed to inherent athleticism that persists across the lifespan [34]. This result also reinforces the need for baseline testing when assessing chronic effects of recurrent concussions. In contrast, younger individuals with prior concussion displayed a greater instability than peers without prior concussion on a subset of balance tests. These differences were observed only during tandem stance assessments, suggesting that challenging individual proprioception may be necessary to detect neurologic effects of recurrent concussions.

It is possible that the group of former professional football athletes in this study lack the necessary number of concussion exposures or severity of concussions to induce long-term cognitive effects. They did not show difficulties with mood symptoms, attention, or motor coordination that represent hallmarks of chronic concussion symptoms. However, these individuals reported an average of five undiagnosed concussions, and undoubtedly experienced numerous subconcussive impacts over their extended professional careers (which lasted, on average, 13 years). They reported a number of concussion-related symptoms on the PCSS, with the greatest number of reports for perceived difficulties involving memory and cognition (which were not assessed through our neurocognitive testing battery).

There were three miRNAs (miR-28-3p, miR-339-3p, and miR-361-5p) that differentiated both former professional football athletes and their peers (Group 1), as well as younger individuals with prior concussion and their peers without prior concussion (Group 2). Levels of these three miRNAs do not show a consistent relationship with balance or reaction time. Instead, they may confer information about the physiologic processes that impact brain function following recurrent concussions. For example, miR-339-3p has been shown to reduce expression of opioid receptors in the hippocampus, an area critical to learning and memory [36]. In the present study, elevated levels of miR-339-3p were observed in both former professional football athletes and individuals with prior concussion. This suggests that miR-339 may contribute to memory difficulty reported by older athletes with recurrent concussions. Levels of miR-28-3p have demonstrated clinical potential as a biomarker for Alzheimer’s disease [37], decreasing in expression with increasing severity of cognitive impairment. Here, we show that salivary levels of miR-28-3p were reduced in former professional football athletes and demonstrated a direct relationship with the number of prior concussions in younger individuals. This pattern may arise as cells increase miR-28-3p expression to regulate remodeling and repair in the early post-concussive period, but later decrease miR-28-3p levels as a result of chronic dysregulation. Although memory performance was not assessed in the older cohort of former professional athletes, over one-third of participants reported subjective difficulty with short-term memory.

To our knowledge, two of these three miRNAs have been identified in prior human studies of traumatic brain injury. Our previous study of salivary miRNA among children and adolescents with mild traumatic brain injury identified a 2-fold decrease in miR-28-3p levels compared to healthy peers [31]. A study of circulating miRNAs among adult patients with chronic hypopituitarism induced by prior severe traumatic injury identified reduced levels of miR-361-5p [38]. The current study is among the first to examine the relationship between miRNAs and chronic concussion symptoms [30,39]. The timing of sample collection relative to the concussion event is likely to have a large impact on miRNA profiles, when comparing these results with prior studies of miRNA levels in concussed individuals. Differences in biofluids of miRNA origin, methods for miRNA isolation, age of participants, and severity of head injury may also account for differences between our investigation and the results of previous studies [27].

Strengths of the current study include the use of two separate case-control groups, recruited from multiple sites, and assessed with standardized measures of symptomology and neurocognitive performance. However, several limitations exist. The sample size for Group 1 is small, limited by the available number of former professional football athletes within the United States population. As noted in the power analysis, this group was adequately powered to identify biomarker candidates, and was used here as an exploratory cohort, with the downstream validation in a larger, external group of participants. It should be noted that there was a significant difference in age and sex between individuals with prior concussion and peers without prior concussion (Group 2). These differences may have impacted comparative symptom reports, neurocognitive results, balance performance, and/or miRNA levels. For this reason, age and sex were used as covariates in our linear regression analysis assessing the relationship between miRNA candidates and number of prior concussions. We acknowledge that functional assessments of balance and neurocognition were performed in a limited subset of participants due to testing capabilities at each clinical site. This may have resulted in selection bias and influenced between groups comparisons for functional assessments. Finally, the case-control design used in Group 1 does not provide an opportunity to assess within-subjects temporal changes in mood, cognition, and motor skills that may occur slowly over time in individuals with prior recurrent concussions. Furthermore, addition of former professional athletes from non-contact sports as controls would strengthen the findings in Group 1. Future studies should address these short-comings to better characterize neurocognitive deficits and gain insights about longitudinal molecular patterns. Parallel measures of established biomarkers of neurodegeneration, such as neurofilament lite, would also strengthen the evidence for miRNA pathophysiology in recurrent concussions.

### Conclusions

The current study provides evidence that saliva miRNAs may provide molecular information about the underlying pathophysiology linking recurrent concussions with chronic symptoms. Here, we identified three concussion-related miRNAs with unique signatures in both younger individuals with prior concussion and older former professional football athletes with a history of recurrent head impacts. Used in conjunction with objective balance tests, non-invasive measurement of these miRNAs may have utility to identify individuals at risk for chronic concussion symptoms.

## 4. Materials and Methods

Ethical approval for this study was provided by an independent institutional review board (Western IRB Study #1271583). Institutional approval was also provided by institutional review boards at the Penn State College of Medicine (STUDY00003729). Written, informed consent was obtained for all participants.

### 4.1. Participants

This study involved two analysis groups: (1) a case-control comparison between 13 former professional American football athletes and 18 age/gender-matched controls (ages 46–89 years); and (2) a cross-sectional study of 310 individuals (ages 7–39 years) who provided a complete history of past concussions. Exclusion criteria for all participants were any concussion in the past 90 days, primary language other than English, pregnancy, active periodontal disease, ongoing neurologic disorder (e.g., epilepsy, multiple sclerosis, cognitive impairment, or movement disorder), drug or alcohol dependency, or active upper respiratory infection. Concussion history, medical history, and a single saliva sample were collected from each participant, and a subset of participants completed computerized balance and neurocognitive testing.

The objective of Group 1 was to identify saliva miRNAs that might be uniquely impacted by recurrent concussions sustained during years of participation in professional American football. Participants included a convenience sample of 13 former professional football athletes, enrolled between September 2018 and January 2020 at the National Football Hall of Fame in Canton, Ohio. Medical characteristics and saliva miRNA from these participants were compared against a control group of 18 individuals without a history of professional sports participation. The control group included healthy volunteers, recruited from the State University of New York (SUNY) Upstate Medical University. The control group was matched to the professional football group by age and sex.

The objective of Group 2 was to determine whether the candidate miRNAs identified in Group 1, would demonstrate relationships with history of past concussions among children and young adults. All 310 participants were enrolled as part of a larger parent study of mild traumatic brain injury between September 2017 and February 2020 at nine institutions: Bridgewater College (Bridgewater, VA, USA; *n* = 20), Colgate University (Hamilton, NY, USA; *n* = 67), the United States Army (Fort Benning, GA, USA; *n* = 29), Marist College (Poughkeepsie, NY, USA; *n* = 25), Penn State University (Hershey, PA, USA; *n* = 112), New York Institute of Technology (NYIT; Old Westbury, NY, USA; *n* = 36), SUNY Upstate Medical University (Syracuse, NY, USA; *n* = 5), Syracuse University (Syracuse, NY, USA; *n* = 9), and Temple University (Philadelphia, PA, USA; *n* = 7). Participants in Group 2 were enrolled at affiliated athletic training clinics and medical clinics. Athletes provided samples in a resting state (prior to sports participation). Participants included athletes involved in football, soccer, lacrosse, mixed martial arts, hockey, crew, and distance running. Concussion history was collected from all Group 2 participants via self-report, and confirmed wherever possible through the electronic medical record. Participants were divided into those with (*n* = 80) or without (*n* = 230) a history of concussion. Among those with a prior concussion, there were 56 participants with a history of a single concussion, and 24 participants with a history of recurrent concussions (i.e., 2 or more concussions).

### 4.2. Measures

Trained research staff collected medical and demographic information for each participant, including sex (male/female), age (years), race (White, Black or African American, Asian, American Indian or Other), body mass index (kg/m^2^), and neuropsychological conditions (presence/absence of attention deficit hyperactivity disorder (ADHD), anxiety, depression). All participants reported presence/absence of any previous concussion, and the number of previous concussions. Computerized balance and neurocognitive assessment was performed for a subset of all participants (Group 1: *n* = 28, 90%; Group 2: *n* = 149, 48%) using the ClearEdge system (Quadrant Biosciences Inc., Syracuse, NY, USA). A subset of Group 1 participants (*n* = 18, 58%) completed standardized olfactory testing using the Brief Smell Identification Test (BSIT). Finally, for the professional football athletes in Group 1 (*n* = 13), current concussion-like symptoms were assessed on a 6-point Likert scale using the Post-Concussion Symptom Scale (PCSS) and total symptom severity (sum of all Likert scores), and total symptom burden (sum of all symptoms >0) were calculated. Former professional football athletes also reported position played, years of playing, presence/absence of chronic symptoms that might result from recurrent concussions (i.e., cognitive impairment, impulsivity, apathy, short-term memory difficulty, and emotional instability), and total number of suspected concussions (both diagnosed and undiagnosed). This last element was incorporated to capture potential brain trauma that may not have been disclosed or identified in an earlier era when concussion reporting and treatment guidelines were less uniform [40].

### 4.3. Saliva RNA

Saliva was collected from each participant in a non-fasting state after oral tap-water rinse, using OraCollect Swabs (DNA Genotek, Ottowa, Canada). All samples were collected between 7 AM and 7 PM, and stored following manufacturer instructions. RNA was isolated from each saliva sample with the miRNeasy Kit (Qiagen, Inc., Germantown, MD, USA) at the Molecular Analysis Core Facility at SUNY Upstate Medical University. This approach enriches for small, non-coding RNA, which are predominantly contained within exosomes, but it does not specifically isolate neuronal exosomes. Quality of RNA was evaluated with the Agilent Technologies Bioanalyzer on the RNA Nanochip. The TruSeq Small RNA Library Prep Kit (Illumina Inc., San Diego, CA, USA) was used to prepare RNA libraries. Sequencing of 50 base pair single end reads was performed on a NextSeq 500 (Illumina Inc., San Diego, CA, USA), at a targeted sequencing depth of 10 million reads per sample. Fastq files were trimmed to remove adapter sequences using Cutadapt (v1.2.1; ([41]) and were aligned using Bowtie (version 1.0.0; ([42]) to miRBase22. Quantification was performed via SamTools (Li et al., 2009; python implementation, pysam 0.15.2) with a custom-built bio-informatics architecture (Human Alignment Toolchain, HATCH; Quadrant Biosciences Inc., Syracuse, NY, USA). The miRNA features with low salivary expression (raw read counts < 10 in > 10% of samples) were excluded. The remaining 264 miRNAs were quantile normalized. Each miRNA feature was scaled through mean centering and dividing by the feature standard deviation.

### 4.4. Statistics

Medical and demographic characteristics were compared between professional football athletes (*n* = 13) and control participants (*n* = 18; Group 1), as well as concussion (*n* = 80) and non-concussion participants (*n* = 230; Group 2) using a two-tailed Student’s *t* test. A one-way analysis of variance (ANOVA) was used to assess differences in balance, neurocognitive, and smell testing among professional football athletes and controls (Group 1), as well as differences in balance and neurocognitive testing among concussion and non-concussion participants (Group 2). To comparative the relative ability of balance assessment, neurocognitive testing, and miRNA profiles for differentiating former professional football athletes and controls, a partial least squares discriminant analysis was used. Next, to identify miRNA candidates whose expression might reflect years of recurrent concussion exposure, levels of the 264 miRNAs with robust salivary levels were compared among professional football athletes and control participants (Group 1), using a non-parametic one-way ANOVA. Next, the miRNA features with significant differences (adj. *p* < 0.05) in Group 1 were compared among individuals with or without a history of concussion (Group 2) via non-parametric one way ANOVA. All ANOVA comparisons were subjected to multiple testing correction with the Benjamini-Hochberg method. Finally, we examined miRNAs with significant differences (adj. *p* < 0.05) in both Group 1 and Group 2 for a relationship with recurrent concussions. For all Group 2 participants, a regression analysis was used to assess the relationship between levels of each miRNA and the number of previous concussions (0: *n* = 230; 1: *n* = 56; 2 or more: *n* = 24), while controlling for age and sex. Physiologic relevance of these miRNA candidates was assessed in DIANA miRPATH v3.0 online software (University of Thessaly, Volos, Greece, [43]) by mapping high confidence messenger RNA targets in Tarbase, and assessing over-representation of Kyoto Encyclopedia of Genes and Genomes (KEGG) pathways through a Fishers Exact Test with Bonferroni correction. Brain relatedness of miRNA candidates was assessed using the Human miRNA Tissue Atlas [44]. All other statistical analyses were performed using Metaboanalyst v4.0 online software (McGill University, Montreal, Quebec, Canada; [45]) and Jamovi v1.1.9 (The jamovi project, 2020, https://www.jamovi.org). A post-hoc power analysis employing measures of standard deviation among salivary miRNAs from our previous concussion studies determined that the sample size in Group 1 provided 80% power to detect a 1.5-fold difference between 264 miRNAs, with a per-gene alpha value of 0.049. The sample size in Group 2 provided 98% power to detect a 1.5-fold difference between the 20 miRNA candidates, with a per-gene alpha value of 0.50.

## Figures and Tables

**Table 1:** Participant characteristics.

	Group 1, Mean (Range)			Group 2, Mean (Range)		
	All (*n* = 31)	Football Athletes (*n* = 13)	Controls (*n* = 18)	All (*n* = 310)	Concussion (*n* = 80)	No Concussion (*n* = 230)
Demographics						
Male sex, No. (%)	31 (100)	13 (100)	18 (100)	208 (67)	61 (76)	147 (64) *
Age (years)	73 (46–89)	73 (66–78)	72 (46–89)	20 (7–39)	21 (10–35)	19 (7–39) *
White race, No. (%)	14 (74)	9 (69)	5 (83)	224 (82)	67 (83)	187 (82)
Medical history						
BMI (kg/m^2^)	28 (20–38)	30 (25–38)	26 (20–34) *	24 (13–40)	25 (13–39)	24 (13–40)
ADHD, No. (%)	0 (0)	0 (0)	0 (0)	24 (8)	8 (10)	16 (7)
Anxiety, No. (%)	0 (0)	0 (0)	0 (0)	18 (5)	4 (5)	14 (6)
Depression, No. (%)	0 (0)	0 (0)	0 (0)	10 (3)	4 (5)	6 (3)
Diagnosed concussions, No. (%)	5 (16)	2 (16)	3 (16)	80 (26)	80 (100)	0 (0) *
No. diagnosed concussions	0.3 (1–5)	0.4 (1–5)	0.2 (0–1)	0.4 (0–7)	1.5 (1–7)	0 (0) *
Undiagnosed concussions, No. (%)	NA	13 (100)	NA	NA	NA	NA
No. undiagnosed concussion	NA	5 (1–25)	NA	NA	NA	NA
Time since last concussion (years)	NA	45 (38–56)	NA	NA	1 (0–7)	NA
Professional football career	NA	13 (2)	NA	NA	NA	NA
PCSS burden	NA	10 (3–21)	NA	2 (0–22)	2 (0–22)	2 (0–19)
PCSS severity	NA	19 (3–78)	NA	4 (0–91)	5 (0–91)	3 (0–42)
Sample collection time (24 h clock)	12 (8–18)	13 (10–18)	11 (8–13)	14 (7–19)	14 (7–18)	12 (7–19) *

* denotes *p* < 0.05 between groups on two-tailed Student’s *t* test. Abbreviations: not available/applicable (NA).

**Table 2:** Functional measures of balance, neurocognition, and olfaction.

	Group 1, Mean (SD)			Group 2, Mean (SD)		
	All (*n* = 28)	Football Athletes (*n* = 12)	Controls (*n* = 16)	All (*n* = 149)	Concussion (*n* = 37)	No Concussion (*n* = 112)
Balance						
TLEO	72 (14)	83 (5)	63 (12) *	85 (3)	85 (3)	85 (3)
TLEC	69 (12)	77 (9)	63 (11) *	84 (4)	84 (4)	84 (3)
TSEO	65 (18)	65 (22)	64 (12)	84 (4)	83 (5)	85 (4) *
TSEC	48 (24)	48 (26)	48 (21)	82 (8)	79 (9)	82 (7) *
TLEOFP	61 (20)	69 (18)	54 (19)	86 (7)	85 (7)	86 (6)
Neurocognition						
SRT1	156 (40)	159 (41)	155 (39)	191 (24)	188 (22)	192 (25)
SRT2	152 (36)	148 (39)	155 (32)	186 (23)	185 (22)	186 (23)
PRT	79 (13)	76 (10)	81 (14)	100 (13)	99 (12)	100 (13)
GNG	94 (15)	91 (12)	96 (17)	121 (13)	121 (13)	121 (13)
Olfaction						
BSIT	69 (23)	63 (23)	82 (17)	NA	NA	NA

Note that balance, neurocognition, and olfaction assessments were available for only a subset of participants in Groups 1 and/or 2. * denotes *p* < 0.05 between groups on two-tailed Student’s *t* test. Note that higher scores indicate superior performance on respective tasks. Abbreviations: not available (NA); two-legs, eyes open (TLEO); two-legs, eyes closed (TLEC); tandem stance, eyes open (TSEO); tandem stance, eyes closed (TSEC); two-legs, eyes open on foam pad (TLEOFP); spontaneous reaction time, trial 1 (SRT1); spontaneous reaction time, trial 2 (SRT2); procedural reaction time (PRT); go-no-go (GNG); Brief Smell Identification Test (BSIT).

**Table 3:** Physiologic targets of candidate miRNAs.

KEGG Pathway	*p*-Value	#Genes	#miRNAs
Adherens junction	3.01 × 10^−17^	20	3
ECM-receptor interaction	2.5 × 10^−07^	9	3
Bacterial invasion of epithelial cells	4.3 × 10^−06^	16	3
Hippo signaling pathway	9.6 × 10^−06^	15	3
Protein processing in endoplasmic reticulum	6.2 × 10^−05^	25	3
Proteoglycans in cancer	4.2 × 10^−4^	24	3
Lysine degradation	9.3 × 10^−4^	7	2
Cell cycle	4.8 × 10^−3^	19	3

Abbreviations: Kyoto Encyclopedia of Genes and Genomes (KEGG).
